# Draft Genome Sequences of Four Nosocomial Methicillin-Resistant *Staphylococcus aureus* (MRSA) Strains (PPUKM-261-2009, PPUKM-332-2009, PPUKM-377-2009, and PPUKM-775-2009) Representative of Dominant MRSA Pulsotypes Circulating in a Malaysian University Teaching Hospital

**DOI:** 10.1128/genomeA.00103-12

**Published:** 2013-01-31

**Authors:** Hui-Min Neoh, Zeti-Azura Mohamed-Hussein, Xin-Ee Tan, Raja Mohd Fadhil B Raja Abd Rahman, Salasawati Hussin, Noraziah Mohamad Zin, Rahman Jamal

**Affiliations:** a Medical Molecular Biology Institute (UMBI); b Center of Bioinformatics Research, Institute of Systems Biology (INBIOSIS); c School of Biosciences & Biotechnology, Faculty of Science & Technology; d Department of Medical Microbiology and Immunology, Faculty of Medicine; e Programme of Biomedical Science, School of Diagnostic and Applied Health Sciences, Faculty of Health Sciences, Universiti Kebangsaan Malaysia, Selangor, Malaysia

## Abstract

Here, we report the draft genome sequences of four nosocomial methicillin-resistant *Staphylococcus aureus* strains (PPUKM-261-2009, PPUKM-332-2009, PPUKM-377-2009, and PPUKM-775-2009) isolated from a university teaching hospital in Malaysia. Three of the strains belong to sequence type 239 (ST239), which has been associated with sustained hospital epidemics worldwide.

## GENOME ANNOUNCEMENT

Genotyping using pulsed-field gel electrophoresis (PFGE) of methicillin-resistant *Staphylococcus aureus* (MRSA) strains circulating in our university teaching hospital revealed the presence of a dominant clone (designated pulsotype B), together with a pulsotype C, whose PFGE profile is only a one-band difference from that of pulsotype B (Xin-Ee Tan et al., submitted for publication). To understand better the biology and evolution of these two strains, we selected two representative strains from each pulsotype (B and C) for whole genome sequencing. The strains PPUKM-261-2009 and PPUKM-377-2009 are representative of pulsotype B, while PPUKM-332-2009 and PPUKM-775-2009 are representative of pulsotype C. Staphylococcal cassette chromosome *mec* (SCC*mec*) typing (1) results revealed that PPUKM-261-2009, PPUKM-332-2009, and PPUKM-775-2009 are of SCC*mec* type III-SCC*mercury*, which harbors *ccrA3B3*, *ccrC*, and class A *mec* complex, while PPUKM-377-2009 is of SCC*mec* type IV, harboring *ccrA2B2* and class B *mec* complex. Multilocus sequence typing (MLST) results (2) showed that PPUKM-261-2009, PPUKM-332-2009, and PPUKM-775-2009 belong to sequence type 239 (ST239), which is widely associated with hospital epidemics (3) and has been reported to be the dominant clone in another Malaysian hospital (4). Conversely, PPUKM-377-2009 is from a new sequence type that has a close match to ST1178.

Whole genome sequencing for all strains was performed with an Ion personal genome machine (PGM) sequencer (Life Technologies, Carlsbad, CA). After sequencing, reads were filtered and trimmed prior to *de novo* assembly using Genome Sequencer (GS) assembler version 2.70. Noncoding rRNAs and tRNAs were predicted using RNAmmer 1.2 server and tRNAscan-SE v1.23 programs, respectively. The protein-coding gene models were predicted using the *ab initio*-based gene predictors Glimmer v3.02 and Genemark.hmm v2.10b, while gene annotations were carried out by BLASTp searched against the NCBI nonredundant (NR), Swiss-Prot, Gene Ontology (GO), and Kyoto Encyclopedia of Genes and Genomes (KEGG) databases. As validation, the assembled sequences were compared with the MRSA reference genomes of N315 (carrying the *ccrA2B2* gene) (5) and TW20 (carrying the *ccrA3B3* gene) (6).

For the *de novo* assembly, 638,248 to 923,828 clean reads spanning 115,836,594 to 180,374,605 bases were assembled and aligned. Ninety-seven to 125 contigs were generated, which totaled 2,956,301, 3,019,328, 2,851,702, and 3,000,990 bp for PPUKM-261-2009, PPUKM-332-2009, PPUKM-377-2009, and PPUKM-775-2009, respectively, with >95% (96.57%, 96.65%, 95.1%, and 96.12%, respectively) coverage for all assemblies when compared against reference genomes. The G+C contents were similar for all 4 strains (32.6% for PPUKM-261-2009, PPUKM-332-2009, and PPUKM-775-2009; 32.7% for PPUKM-377-2009). About 2,000 proteins were annotated for all strains using Swiss-Prot, while GenBank NR annotation predicted a larger number of proteins (>2,800) for each strain, which requires further validation through experiments (7). All strains had 2 copies of 5S rRNAs and 1 copy each of 16S and 23S rRNAs, whereas the numbers of predicted tRNAs were in the range of 28 to 53. Gene ontology (a functional classification) showed similar distributions for all 4 strains, which is owed to their close relatedness as visualized through PFGE typing. Further studies of the sequenced genomes are currently under way.

### Nucleotide sequence accession numbers. 

The draft sequences of PPUKM-261-2009, PPUKM-332-2009, PPUKM-377-2009, and PPUKM-775-2009 have been deposited in DDBJ/EMBL/GenBank with the accession no. AMRB00000000, AMRC00000000, AMRD00000000, and AMRE00000000, respectively. The versions described in this article are the first versions: AMRB01000000, AMRC01000000, AMRD01000000, and AMRE01000000.
